# Supplementary material for: Head-to-Head Comparison of Neck 18F-FDG PET/MR and PET/CT in the Diagnosis of Differentiated Thyroid Carcinoma Patients after Comprehensive Treatment
Source: Cancers (Basel). 2021 Jul 9;13(14):3436. doi: 10.3390/cancers13143436 (PMC8307331; doi:10.3390/cancers13143436)
Supplement: Supplementary file 1 [file cancers-13-03436-s001.zip › cancers-1244279-supplementary.pdf]

Supplementary Materials:

Type of the Paper (Article)

Head-to-head comparison of neck ^{18}F-FDG PET/MR and PET/CT in the diagnosis of differentiated thyroid carcinoma patients after comprehensive treatment

Yangmeihui Song[1,2#], Fang Liu[1,2#], Weiwei Ruan[1,2], Fan Hu[1,2], Muhsin H. Younis[3], Zairong Gao [1,2], Jie Ming [4], Tao Huang [4], Weibo Cai[3,5], Xiaoli Lan[1,2,*]

1 Department of Nuclear Medicine, Union Hospital, Tongji Medical College, Huazhong University of Science and Technology
2 Hubei Province Key Laboratory of Molecular Imaging
3 Departments of Radiology and Medical Physics, University of Wisconsin-Madison, Madison, WI, USA
4 Department of Breast and Thyroid Surgery, Union Hospital, Tongji Medical College, Huazhong University of Science and Technology
5 University of Wisconsin Carbone Cancer Center, Madison, WI, USA
# Yangmeihui Song and Fang Liu contributed equally to this work.
* Correspondence: xiaoli_lan@hust.edu.cn

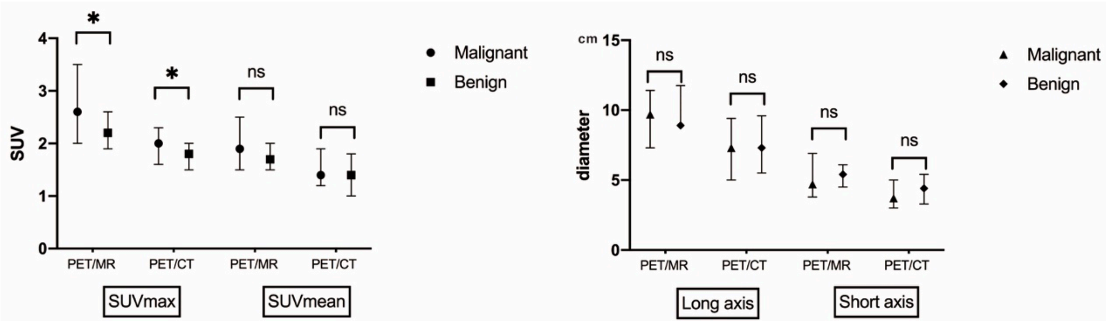

Figure S1. SUVs, diameters of malignant and benign lymph nodes detected by both PET/MR and PET/CT; ns Represents no significance; * Represents the p-value <0.05

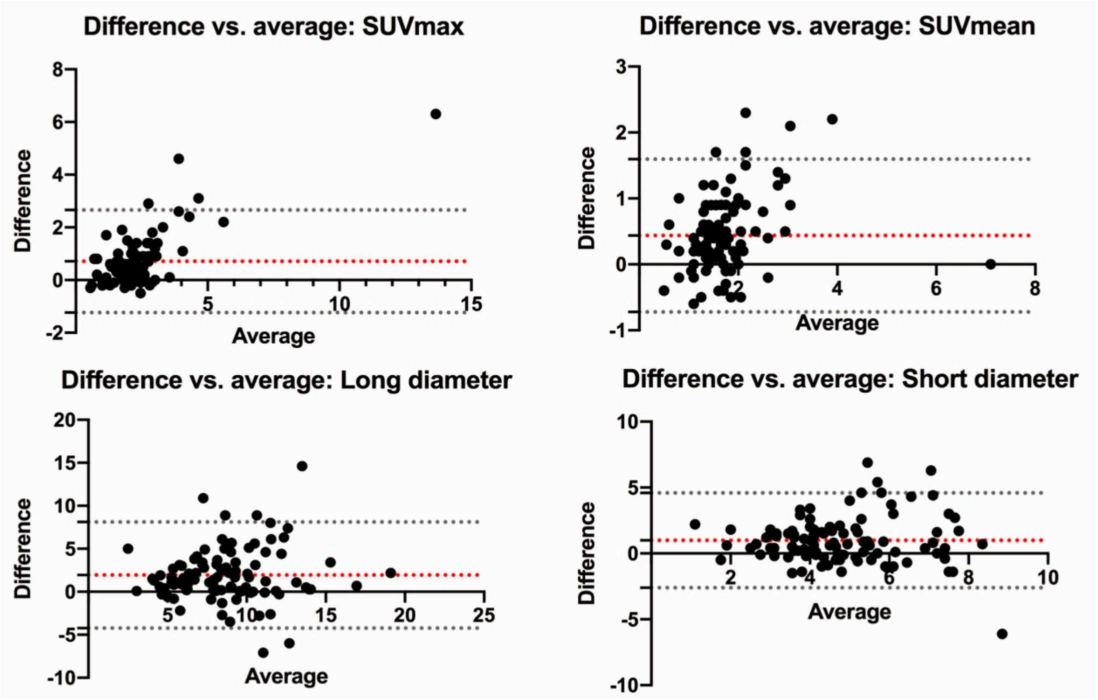

Figure S2. The Bland-altman analysis of SUVmax, SUVmean and diameter measurements provided in two modalities

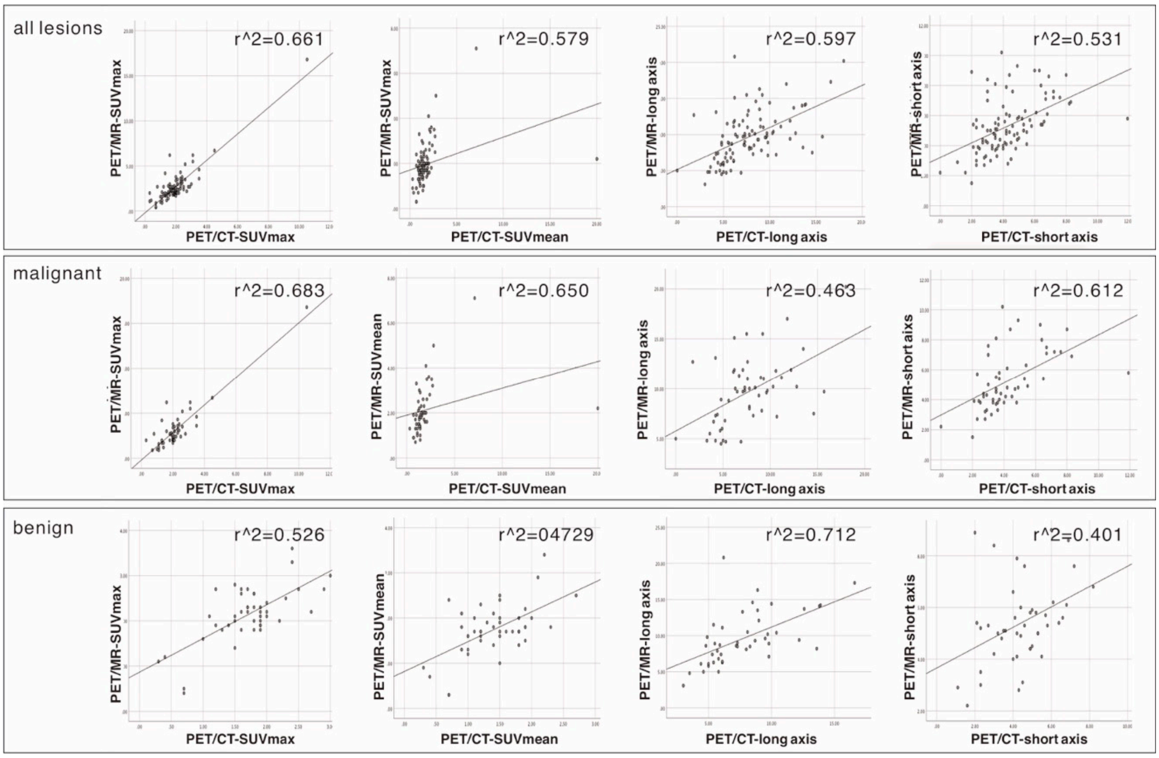

Figure S3. Correlation of SUVs and lymph node diameters measured on PET/MR and PET/CT
